# Recent advances in bioprinting techniques: approaches, applications and future prospects

**DOI:** 10.1186/s12967-016-1028-0

**Published:** 2016-09-20

**Authors:** Jipeng Li, Mingjiao Chen, Xianqun Fan, Huifang Zhou

**Affiliations:** Department of Ophthalmology, Ninth People’s Hospital, Shanghai Jiao Tong University School of Medicine, Shanghai, 200011 People’s Republic of China

**Keywords:** Tissue engineering, 3D bioprinting, Artificial organs

## Abstract

Bioprinting technology shows potential in tissue engineering for the fabrication of scaffolds, cells, tissues and organs reproducibly and with high accuracy. Bioprinting technologies are mainly divided into three categories, inkjet-based bioprinting, pressure-assisted bioprinting and laser-assisted bioprinting, based on their underlying printing principles. These various printing technologies have their advantages and limitations. Bioprinting utilizes biomaterials, cells or cell factors as a “bioink” to fabricate prospective tissue structures. Biomaterial parameters such as biocompatibility, cell viability and the cellular microenvironment strongly influence the printed product. Various printing technologies have been investigated, and great progress has been made in printing various types of tissue, including vasculature, heart, bone, cartilage, skin and liver. This review introduces basic principles and key aspects of some frequently used printing technologies. We focus on recent advances in three-dimensional printing applications, current challenges and future directions.

## Background

The loss or failure of organs and tissues is a difficult and costly problem in healthcare. The limited supply of organs globally [1] has motivated research on tissue engineering, particularly the design of a cell-scaffold-microenvironment to promote the regeneration of various types of tissue, e.g., skin [2], cartilage [3], bone [4], tendon [5] and cardiac tissue [6].

Scaffolds are considered the key element for tissue regeneration because they provide the necessary mechanical support and a physical structure for the transplanted cells to attach, grow and maintain their physiological functions. A suitable scaffold, such as a bone scaffold for tissue engineering, must have favorable biocompatibility or cytocompatibility to provide a surface for cells to adhere, proliferate, differentiate and secrete extracellular matrix (ECM). ECM contains abundant bioactive molecules, including glycosaminoglycans, collagen, fibronectin and cytokines. Pore size and interconnectivity also play important roles in cell adhesion and migration, vascularization and new tissue ingrowth [7–11]. Thus, a fully satisfactory scaffold must simultaneously support the growth of different cell types and tissues, each with specific mechanical properties, chemical gradients, cell populations, and geometric structures. However, conventional fabrication methods [12, 13] used for manufacturing three-dimensional (3D) scaffolds, such as electrospinning, fiber deposition, freeze-drying, gas foaming, and salt leaching, lack precise control of internal structural features and topology. Therefore, techniques for the accurate fabrication of multifunctional scaffolds are needed. These complex design constraints limit the effectiveness of many current traditional methods, particularly when attempting to repair clinically relevant injuries, organs, and other complex tissues.

**Fig. 1:**
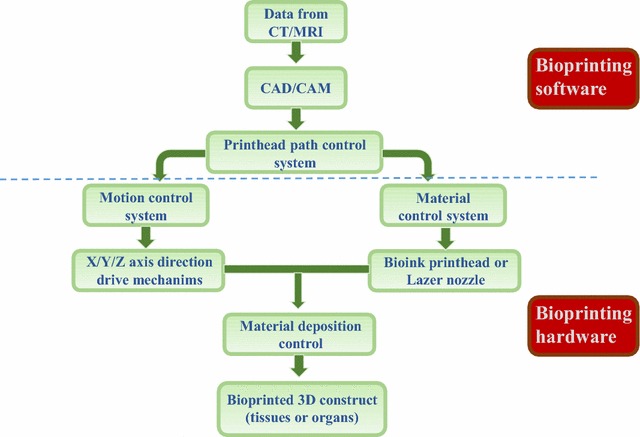
General 3D bioprinting technical route

Additive manufacturing (AM) technology is increasingly recognized as a potential solution for constructing complex interfacial tissue engineering scaffolds. AM forms complex 3D biocompatible structures via automated deposition of biological substances on a substrate using computer-aided design/computer-aided manufacturing (CAD/CAM) technology. The working principle of AM is that objects can be created by adding material in a layer-by-layer manner, in contrast to conventional machining, which removes material in a subtractive manner [14]. 3D bioprinting is an important type of the AM technology which focus on printing bioactivity substance. Bioprinting can control the shape, size, internal porosity and interconnectivity of a tissue-engineering scaffold (Fig. 1). Moreover, some types of bioprinting technology are capable of fixed-point deposition of cells and biomolecules, such as DNA, Polycose® and cytokines. Micro-tissues, micro-organs or mimetic extracellular matrix (mECM) can provide researchers with an effective strategy to study disease progression [15] and mechanisms of drug action [16, 17], in addition to applications in tissue or organ transplantation [18, 19].

3D bioprinting technology has attracted increasing attention based on its immense potential in the manufacture of tissue-engineering compounds. This review focuses on the key elements of 3D bioprinting technology used to fabricate very precise scaffolds and the applications of printing-specific modeling used in patient preoperative planning and the production of artificial tissues or organs for implantation. The article also discusses challenges and potential future directions.

## Bioprinting technologies and their applications

We have summarized 3D printing techniques frequently utilized for scaffold fabrication, cell behavior studies and tissue repair (Table 1).

**Table 1:** Characteristics of bioprinting processes

	Biomaterials	Cell viability/resolution	Bioprinting speed	Cost	Advantages	Disadvantages	References
Inkjet-based bioprinting	Low-viscosity suspension of living cells; biomolecules; growth factors	~90 % 20–100 µm	Fast (<10,000 droplets/s)	Low	Wide availability; low cost; high resolution; high printing speed; ability to introduce concentration gradients in 3D constructs	Poor vertical structure clogging characteristics; thermal and mechanical stress to cells; limited printable materials (liquid only)	[24, 28–31]
Pressure-assisted bioprinting	Hydrogel; melt; cells; proteins and ceramic materials; solutions, pastes, or dispersions of low to high viscosity; PLGA; tricalcium phosphate (TCP); collagen and chitosan; collagen-alginate-silica composites coated with HA; and agarose with gelatin	40–80 % 200 µm	Slow	Medium	Numerous materials that can be printed with any dimensions; mild conditions (room temperature); use of cellular spheroids; direct incorporation of cells; and homogenous distribution of cells	Limited mechanical stiffness; critical timing of gelation time; specific matching of the densities of the material and the liquid medium to preserve shapes; low resolution and viability	[33, 102–104]
Laser-assisted bioprinting	Hydrogel, media, cells, proteins and ceramic materials of varying viscosity	>95 % >20 µm	Medium	High	Nozzle-free, noncontact process; cells are printed with high activity and high resolution; high control of ink droplets and precise delivery	High cost; cumbersome and time consuming; requires a metal film and thus is subject to metallic particle contamination	[39–41]
Stereolithography	Light-sensitive polymer materials; curable acrylics and epoxies	>90 % ~1.2–200 µm	Fast (<40,000 mm/s)	Low	Solid freeform and nozzle-free technology; highest fabrication accuracy; compatibility with an increasing number of materials; light-sensitive hydrogels can be printed layer-by-layer	Applicable to photopolymers only; lack of biocompatible and biodegradable polymers; harmful effects from residual toxic photo-curing reagents; possibility of harm to DNA and human skin by UV	[45]

### Inkjet-based bioprinting

**Fig. 2:**
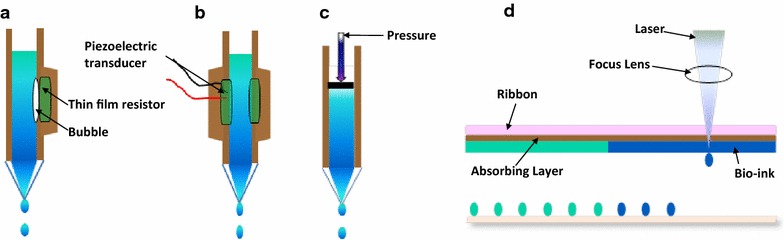
Common types of bioprinting methods. **a** Thermal inkjet-based bioprinting technology utilizes an electric current pulse that impulses the thin film resistor, then generates bubbles that create a pressure pulse that propels the ink droplet onto the substrates. **b** A piezoelectric transducer creates a pulse that creates transient pressure, resulting in droplet ejection. **c** Pressure-assisted bioprinting uses solutions, pastes, or dispersions as biomaterials, which are extruded by pressure in the form of a continuous filament through a microscale nozzle orifice or a microneedle. **d** Laser-associated bioprinting consists of three parts: a pulsed laser source, a ribbon and a receiving substrate. The lasers irradiate the ribbon, causing the liquid biological materials to evaporate and reach the receiving substrate in droplet form

Inkjet-based bioprinting is a type of bioprinting technology based on the conventional inkjet printing process with desktop inkjet printers. It is a noncontact printing process that deposits precise picoliter droplets of “bioink” onto a hydrogel substrate or culture dish under computer control. The common methods can be further classified into thermal and piezoelectric actuator methods based on the droplet actuation mechanism [20]. In thermal technology, ink droplets are generated by heating so that an inflated bubble forces the ink out of the narrow nozzle and onto the substrates (Fig. 2a). The localized temperature can reach hundreds of degrees in only a few microseconds to generate pulse pressure [21]. This technology is inexpensive and has been used broadly [22, 23]. However, the droplets prepared using the thermal technology are mixed, unordered and unequal in size. Because of frequent nozzle blockages, smooth printing is difficult. Shear and thermal stress also affect the viability of the cellular and protein inks [24]. In piezoelectric technology, drops are generated by the transient pressure from piezoelectric actuator (Fig. 2b). In contrast to thermal technology, the piezoelectric method does not use heat and does not cause orifice clogging, allowing droplets to remain directional with regular and equal size [25, 26]. However, piezoelectric technology can cause damage to the cell membrane and cell lysis if used too frequently [27]. Greater than 90 % viability has been reported for piezoelectrically deposited mammalian cells, including human osteoblasts, fibroblasts, and bovine chondrocytes [26].

Scientists have made great progress in patterning molecules, cells and organs by inkjet printing. Molecules such as DNA have been successfully printed [28], facilitating studies of cancer pathogenesis and treatment. In addition, thermal inkjet printing has been demonstrated to be biocompatible with Chinese hamster ovary (CHO) cells and rat embryonic motoneurons [29]. Less than 8 % of CHO cells were lysed in the printing process, indicating that mammalian cells can be successfully printed by inkjet bioprinting and retain their functions, with good prospects for creating living tissue structures or organs. Further developments in bioprinting technology have resulted in advancements in the printing of functional blood vessels and heart valves. In 2015, Jana and Lerman studied the bioprinting of cardiac valves to solve clinical transplantation shortages. Cardiac constructs are complex and important, particularly the four valves of the heart. Although heart valves have been successfully printed, the functional requirements of elasticity and physiological conditions remain to be fulfilled [24]. Similarly, in 2014, Duan B examined printing of blood vessels and observed drawbacks similar to those observed for bioprinted heart valves. Hydrogels, the main biomaterials used in inkjet bioprinting, are too soft to withstand normal physiological conditions [30, 31]. Thus, to successfully print organs that maintain good biological function in vivo, new biological materials that are more suitable for the human body must be developed to match the mechanical and biological properties of native organs.

### Pressure-assisted bioprinting and its applications

Pressure-assisted bioprinting (PAB) is based on extrusion to create desired 3D patterns and constructs. The biomaterials used for printing are usually solutions, pastes, or dispersions [32] that are extruded by coordinating the motion of pneumatic pressure or plunger- or screw-based pressure in the form of a continuous filament through a microscale nozzle orifice or a microneedle onto a stationary substrate. After layer-by-layer application, complete 3D patterns and constructs are eventually formed (Fig. 2c).

The advantages of PAB technology include room temperature processing, direct incorporation of cells and homogenous distribution of cells. PAB has been applied to the printing of cell and organs with confirmed retention of activity. Bioprinted cells include mouse pre-osteoblasts, human mesenchymal stem cells, endothelial cells, and osteogenic progenitors. Bioprinted cells have been used to repair ovine calvarial defects [33]. The feasibility of multicellular bioprinted constructs incorporating goat multipotent stromal cells (MPSCs) and endothelial progenitor cells with retention of heterogeneous cell organization in the subcutaneous tissue of immunodeficient mice and production extracellular matrix has been demonstrated. The multipotent stromal cells in the multicellular bioprinted constructs differentiated into bone structures, and the endothelial progenitor cells differentiated into blood vessels. These results support the ability of multicellular bioprinted grafts to retain activity in vivo [34, 35].

### Laser-assisted bioprinting

Laser-assisted bioprinting (LAB) uses a laser as the energy source to deposit biomaterials onto a substrate. This technique usually consists of three parts: a pulsed laser source, a ribbon coated with liquid biological materials that are deposited on the metal film, and a receiving substrate [24]. The lasers irradiate the ribbon, causing the liquid biological materials to evaporate and reach the receiving substrate in droplet form. The receiving substrate contains a biopolymer or cell culture medium to maintain cellular adhesion and sustained growth after transfer of cells from the ribbon (Fig. 2d). LAB mainly uses nanosecond lasers with UV or near-UV wavelengths as energy sources to print hydrogels, cells, proteins and ceramic materials [36, 37]. The resolution varies from pico- to micro-scale features and is affected by many factors: the thickness of the biological materials on the film, their rheological properties, the energy of the laser pulse, the wettability of the substrate, and the printing speed and organization of the structure [38, 39].

Researchers have demonstrated the feasibility of using laser-based technology to print cells, for example, human dermal fibroblasts, mouse C2C12 myoblasts, bovine pulmonary artery endothelial cells (BPAECs), breast cancer (MCF-7) cells and rat neural stem cells [39–41]. In 2013, Michael et al. [42] successfully created Graftskin skin substitutes by utilizing LAB technology, a landmark event in the field of laser-assisted bioprinting. Fibroblasts and keratinocytes were used as the cell sources to fabricate the skin constructs, which were subsequently transplanted into the skin folds of nude mice to perform an in vivo evaluation. Eleven days later, the graft adhered well to the tissue around the skin wound. The keratinocytes proliferated and differentiated well and grew to the skin stratum corneum and basal layer. Compared with other bioprinting technologies, LAB has unique advantages, including a nozzle-free, non-contact process, the printing of cells with high activity and high resolution, and the control of ink droplets and precise delivery characteristics. Ink bubble dynamics, shear stress and laser pulse energy all play important roles in the bioprinting process [43–45].

### Stereolithography

Stereolithography (STL) technology is a solid freeform, nozzle-free technology that was developed in the late 1980s [46]. A liquid, photo-sensitive polymer formulation is solidified upon illumination. STL uses digital micromirror arrays to control the light intensity to polymerize light-sensitive polymer materials. This technique is mainly applied to fabricate structures from curable acrylics and epoxies. The number of photocrosslinkable polymers is increasing, and multiple resins can be used for one structure [32]. Digital light projection controls the printing process in this top-down system. Compared with other solid freeform techniques, STL has the highest fabrication accuracy, and an increasing number of materials can be used in this process. Furthermore, STL can print light-sensitive hydrogels layer-by-layer, and the total printing time depends only on the thickness of the structure [47].

However, in addition to these advantages, there are numerous restrictions, such as the lack of proper biocompatible and biodegradable polymers, harmful effects from residual toxic photocuring reagents, the inability to completely remove the supporting structure and the inability to form horizontal gradients in the constructs [46]. UV-sensitive photoinitiators were once used, although UV is harmful to cellular DNA and causes skin cancer. In 2015, Wang [47] investigated the use of customized visible light in a STL system, including a commercial beam projector and bioinks, a mixture of PEGDA, methacrylated gelatin (GelMA), and eosin Y-based photoinitiator. They first described the detailed protocol of the visible light-based STL system and revealed the necessity of an infrared ray (IR)-filtering water filter to the system. Their experimental results with NIH 3T3 cells demonstrated that this system with customized visible light could support the bioprinting of visible light-curable hydrogels with 50-μm resolution and high cell viability (∼85 %) for at least 5 days.

## Key points of bioprinting

The goal of tissue engineering is to fabricate 3D artificial tissues or organs composed of a scaffold, cells and a microenvironment that mimics the real environment of the human body. As a highly effective and accurate method to fabricate artificial tissue in vitro, printing achieves these three necessary components. This section will discuss the applications and limitations of the materials used in bioprinting, including biomaterials, cells and cell factors.

### Parameters of biomaterials

In general, biomaterials can be categorized into a large variety of hydrogel, metallic, ceramic, polymeric and composite materials. The physical characteristics of biomaterials determines the optimal printing type. For example, low-viscosity materials are more attractive for bioprinting because cells can grow well in the low-pressure environment [48]. Other material properties, such as pore size and interconnectivity, also influence the encapsulated cells [49].

#### Biocompatibility

The biocompatibility of biomaterials is the first parameter to be considered when fabricating scaffolds and significantly limits the number of suitable materials. Scaffold materials must accommodate the encapsulated cells and the recipient’s body. Thus, the implant must be cytocompatible and support cell growth, attachment, proliferation and migration but safe for the host and not cause severe inflammation or immunologic rejection.

Hydrogels are attractive materials for bioprinting because they are an enormous three-dimensional network of polymer chains holding a mass of water. For the processing of physical hydrogels, a polymer network is expected to form from the physical junctions between hydrogel macromolecules. The use of some photo-initiators and monomers during hydrogel crosslinking affects cell viability depending on radical concentration and the length of exposure [50]. However, more complex, functional and biocompatible hydrogels can be fabricated using bioprinting technology. Wüst et al. [51] reported the use of different amounts of HA to print a tunable alginate-gelatin hydrogel composite with a two-step crosslinking procedure. Human mesenchymal stem cells (hMSCs) were subsequently mixed with the hydrogel, and cell viability was detected. Approximately 85 % of the cells were still viable after 3 days of in vitro culture. This experiment demonstrated that adding HA to the hydrogel in different concentrations enhances mechanical properties to match hard tissue reconstruction with no reduction in cell viability.

#### Porosity and interconnectivity

Pore shape, volume, size and geometry all directly affect the behavior of cells after adhesion to the scaffold. Different pore sizes in matrices can affect extracellular matrix development and are strictly correlated with cellular organization, collagen I assembly, and mineralization [52]. Porosity and interconnectivity play important roles in the ingrowth of surrounding tissues. Open and interconnected pores can allow oxygen and nutrients to be transported into the interior and eliminate the waste generated by cellular metabolism.

Domingos et al. [53] performed a systematic analysis of 3D-printed scaffolds to determine the effects of pore size and geometry on hMSC viability. Three different filament distances (550, 650 and 750 μm) and filament patterns (0°*/*90°, 0°*/*60°*/*120° and 0°*/*45°*/*90°*/*135°) were designed to obtain complex internal geometries. Cells encapsulated in the structure with larger filament distances and fewer deposition angles behaved more actively. The results indicated the following: (1) cell adhesion, viability and proliferation were strongly influenced by the pore size and shape, whereby large quadrangular pores enhanced hMSC viability and proliferation; (2) cell morphology did not seem to be affected by pore topology, as demonstrated by the investigation of the shape factor.

#### Mechanical properties

Physical parameters are an indispensable part of tissue engineering scaffolds, particularly for the regeneration of hard tissues, such as bones and cartilages. Appropriate mechanical strength matching that of natural bones is very important. When artificial bones with high elastic moduli are implanted in situ, they may produce stress shielding and hinder new bone formation. The mechanical properties of human cortical and cancellous bone are generally described as in Table 2.

**Table 2:** Characteristics of human cortical and cancellous bones

Bone type	Porosity (%)	Pore size (μm)	Compressive strength (MPa)	Young’s modulus (GPa)
Cortical bone	3–12	<500	130–225	3–30
Cancellous bone	50–90	500–1000	4–12	0.01–0.5

Ceramics [54–57] such as TCP, CaP, HA and SiO_2_ are widely used in bone tissue engineering because of their excellent mechanical properties, osteoconductivity and comparability with bones. Some examples of bioprinted ceramic materials are listed in Table 3.

**Table 3:** 3D-printed ceramic materials for tissue engineering

Material	Porosity and compressive strength	Biological properties	Printing type	References
SiO_2_/ZnO	32–52 % and 2–10 MPa	Increased mechanical strength and cellular proliferation	Inkjet-based bioprinting	[105]
β-TCP/POC (poly-1,8-octanediol-co-citrate)	45 %	High compressive modulus and good drug delivery performance	Micro-droplet jetting	[106]
CaSiO_3_	70 % and 7 MPa	Enhanced cell attachment and osteogenic activity	3D printing	[100]
CaCO_3_/SiO_2_	34 % and 47 MPa	resulting in improved mechanical properties and good cell affinity	Laser-aided gelling (LAG)	[107]
Sr–Mg doped TCP	4–12 MPa	Increased osteons and, consequently, an enhanced network of blood vessel formation and osteocalcin expression	3D printing	[108]
HA/PVOH (poly(vinyl)alcohol)	55 % and 0.88 MPa	Osteoconduction and osteointegration in vivo	3D printing	[109]
HSP bioceramic (hollow-struts-packed)	65–85 % and ~5 MPa	Significantly improved cell attachment and proliferation; promotion of formation of new bone tissue in the center of the scaffolds	A modified coaxial 3D printing	[110]

Most material components of bioink are derived from current materials used in tissue engineering and limit the application of printed scaffolds. In addition to good biocompatibility, high porosity and matching mechanical properties, the ideal material must have appropriate hydrophilicity, pH neutrality, and degradability without the formation of toxic macromolecules. Future development of manufacturing technology will enable printing of biofunctional scaffolds that perfectly mimic the extracellular matrix to provide cells with a microenvironment for adhesion, proliferation and directional differentiation.

#### Classification and ink formulations

A wide range of biomaterial inks which categorize polymers, ceramics, hydrogels and composites have been developed in the printing technology [58]. Compared to polymer and ceramic, hydrogel inks have received much more attention, and significant progress have already been made to design novel ink formulations. A new 3D bioprinting technique called freeform reversible embedding of suspended hydrogels (FRESH) has been introduced by Feinberg AW [59]. The innovation of this technology is what deposits and crosslinks one kind of hydrogel inks into another that can be considered as a support carrier. It has been successfully applied in the printing of human femurs, branched coronary arteries, trabeculated embryonic hearts and human brains [60].

The lack of diversity in “biomaterial inks” became barriers to the widespread applications of 3D printing. UV light, chemical cross-linking, and high temperatures in the materials machining negatively impact most biologically activities. However, utilizing cellularized matrix gels lacked initial mechanical strength. The balance between structural strength and biocompatible processing is hard to satisfy scientists [61]. In the future, more bioactive and more mechanically stable must be developed to ultimately serve as the “bioink” for bioprinting tissue constructs.

### Bioprinting cells

Cell printing is the key element for the printing of tissue and organs. However, the choice of bioink materials is limited by the stringent printing conditions. The stiffness, functional groups, and surface morphology of biomaterials have a significant impact on cellular behavior. Cells are usually encapsulated in biodegradable hydrogels that mimic a tissue-like environment for building bioprinted ink [62]. The characteristics of hydrogels can protect the inner cells from the shear force generated in the printing process and maintain their bio-functions, such as the self-renewal ability and multi-lineage differentiation potency of stem cells. The cytocompatibility of laser-assisted cell printing technology with cells post-deposition was recently demonstrated, and this technique has been widely used for its high resolution and accuracy in single-cell deposition.

#### Viability of post-printed cells

CHO and embryonic motoneuron cells [29] were first successfully deposited into pre-defined patterns in 2005. That study emphasized the need to study the biocompatibility of the inkjet printing process and the ability to encapsulate cells into bioink. The results were satisfactory, and less than 8 % cell death was observed. Researchers [62] have successfully constructed HepG2-loaded GelMA hydrogels exhibiting high cell viability of greater than 95 % for at least 8 days. These achievements demonstrated the possibility of bioprinting complex, cell-laden hydrogel tissue constructs. Cells embedded in the hydrogels may remain in a non-proliferating state [63]. Neufurth et al. used the calcium salt of polyphosphate (polyp-Ca2^+^-complex) as a second layer on top of an inner sodium alginate hydrogel surface, which strongly promoted cell proliferation and enhanced the hydrogel mechanical strength.

Bioprinted cells maintain their proliferation and differentiation abilities in vitro, an important step in the development of tissue constructs. Lorber et al. [64] reported that adult rat RGCs (retinal ganglion cells) and glia could be successfully printed by a piezoelectric inkjet printing method. No significant differences in cell survival and outgrowth were observed between the non-printed control group and the printed group. Additionally, coating a glial substrate first and then printing RGCs on top enhanced the functional activity of the cells. Future goals include printing other cells of the retina, particularly the light-sensitive photoreceptors, with exciting implications for the printing of a functional retina.

#### Bioprinting stem cells

Stem cells, including embryonic stem cells (ESCs), BMSCs and ASCs, can be printed and patterned by precise deposition of picoliter (pl) volumes of fluid or laser-aided accurate localization. An important concern in stem cell printing is that the activity of stem cells, including proliferation and pluripotency, may change during the printing process. Levato et al. [65] encapsulated MSCs in gelatin methacrylamide-gellan gum bioinks that combined bioprinting with microcarrier technology. This bioprinting approach allowed cells to be deposited internally with 90 % viability after 3 days, and the cells were induced to osteogenic differentiation, with increased expression of bone markers such as ALP and OCN. MSCs were submerged in perfluorotributylamine (C12F27N) as a hydrophobic high-density fluid to be printed in the desired shape [66]. A printed vascular bifurcation maintained its shape and dimensions for more than 6 months.

In the laser-assisted field, MSCs [67] were printed based on laser-induced forward transfer (LIFT) for the construction of scaffold-free autologous grafts. The seed cells survived the complete printing procedure and maintained their ability to proliferate and continue differentiating into the osteogenic lineage. Laser-induced jet formation and jet dynamics were explored using time-resolved imaging [45]. Slow jets were unperturbed, with increased stability and retention of stem cells with very high viability and high resolution. Ultraviolet (UV) light used in traditional laser-assisted printing technology might be damaging to the cellular DNA. Lin et al. [68] reported a visible light-based projection STL system that successfully incorporated hASCs in hydrogel scaffolds.

#### Single-cell patterning

As bioprinting technology has developed, single-cell deposition onto two-dimensional (2D) and into 3D environments has been used to explore cell behavior and monitor responses to growth, physical stimulation, cytokines and metabolite factors at the cellular level. A single-cell throughput system was also utilized to explore stem cell characteristics [69]. Based on the microdroplet throughput, the authors isolated and patterned single cells from heterogeneous cell suspensions. The printed cells maintained high viability of greater than 95 %, and 11 stem cell markers (including Kit and Notch1) were collected and analyzed from the genomic information. In Ma et al.’s work [70], researchers utilized a laser-patterned method to control MSC alignment to create a parallel-aligned morphology and studied cardiogenic differentiation and contractile function. Single-cell arrays have been widely utilized in neural networks. Dinh et al. [71] constructed compartmentalized brain models with single-cell precision based on microfluidic methods.

Significant progress has been made in controlling printing conditions to ensure minimal damage to cells and adequately mimic the extracellular environment. However, to print tissue structures, different cell types must be placed in specific locations, and stem cell differentiation must be controlled to produce the desired cell types [14]. Organ printing remains a long-term goal, and micro-organ printing has more potential in clinical applications. For example, islet cells with secretory capacity account for only 2 % of pancreas cells. Printing these functional cells and reintroducing them into patients may allow patients without a complete pancreas to continue producing insulin.

### Extracellular microenvironment

The extracellular microenvironment or niche provides various stimuli, such as physical, chemical and biological factors, to direct cell adhesion, proliferation and differentiation. Obvious effects on cell behavior have been confirmed by directly designing the surface morphology of scaffolds [72].

Biological molecules, including proteins and nucleic acids, have been successfully deposited with bioprinting technologies such as inkjet printing [14]. An advantage of inkjet printing is the ability to control the concentration gradient of internal ingredients using different bioink densities [73].

Growth factors such as BMP2, epidermal growth factor (EGF) and fibroblast growth factor 2 (FGF-2) have also been measured by molecular patterning. To print 3D artificial tissues, studies of 2D molecular arrays may provide clues about the function of growth factors in their niche.

## Advanced applications of bioprinted tissues and organs

**Fig. 3:**
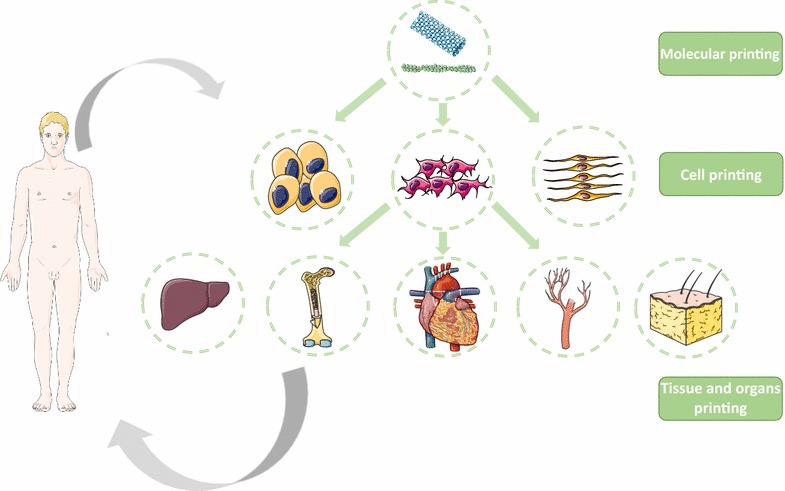
The applications of bioprinting range from the molecular level to organ level

Artificial tissues and organs are printed by depositing cells, biomaterials and molecules layer by layer. Bioprinting has the advantage of good resolution of the input cells. Using this technology, great efforts have been made in developing bioprinting technologies to attempt to print blood vessels [74], hearts [75], bone [76], cartilage [77], kidneys [78], skin [79], nerves and other tissues (Table 4). Figure 3 shows that the bioprinting technology has a wide range of applications from the molecular level such as DNA and protein to organ level.

**Table 4:** Applications and directions of bioprinting organs

Bioprinted tissues and organs	3D printing technology	Applications	Future directions	References
Blood vessels	Inkjet bioprinting	Optimizing vascular geometry and cell viability and function Predicting flow rates, oxygen tension, and the diffusion of molecules in the vascular environment	Improving resolution for printing small vessels Increasing available bioink materials Increasing bioprinting speed	[22, 111]
	Extrusion bioprinting			
	Laser-assisted bioprinting			
Heart	Extrusion-based bioprinting	Printing valvular interstitial cells into scaffolds with high speed and good viability (~100 %) over 21 days Printing hydrogel-based valve-shaped structures	Developing types of materials with good flexibility and elasticity	[31, 77, 83]
	FRESH			
Bone	SLA	Printing scaffolds that provide a framework for cells to attach, proliferate and function and to be integrated with the surrounding tissue Accurately controlling pore geometry, cell viability and mechanical properties	Investigating printed materials with osteoinductive or osteoconductive proteins Triggering vascularization in the repaired region	[112, 113]
	Laser-assisted bioprinting			
Liver	Inkjet printing	Printing biological livers for liver transplantation in patients with liver resection Constructing artificial liver tissue for the detection of drug toxicities and other medical and biological testing	Constructing 3D functional liver tissue with a substantial capillary-like network	[95, 99, 114]
Skin	Inkjet bioprinting	Fabricating skin substitutes to repair skin wounds Studying the pathophysiology of skin diseases	Fabricating more complex human skin models with secondary and adnexal structures Improving LAB technology to achieve automation for bioprinting skin	[42, 93]
	Extrusion bioprinting			
	Laser-assisted bioprinting			

### Blood vessel and heart printing

As the mode of transport for nutrition and metabolic waste, functional blood vessels play important roles in cardiovascular diseases [80] and the construction of artificial organs, particularly these with a rich blood supply. Advances in developmental biology and iconography have enabled significant progress in printing vasculature in vitro. However, due to the unique functions and specific structures of the vasculature in different tissues, creating a vascular system remains a critical, unmet challenge.

Bioprinting enables the fabrication of network structures using hydrogels or other materials as bioink. Bertassoni et al. [81] successfully bioprinted a vascular network with GelMA, which improved metabolic transportation, cellular viability and the formation of endothelial monolayers. Kolesky et al. [82] successfully co-printed multiple materials with cells and vasculature using thermally reversible gelation to fabricate complex tissues.

In the printing of cardiac valves, tissue-engineered valve scaffolds are generally focused on the rebuilding of the aortic valve [24]. Researchers have performed extensive studies of printing aortic valve structures with hydrogels [30, 31, 83]. Cell-laden, valve-shaped structures were printed with a high cell survival rate (greater than 90 %). However, bioink materials are deficient in flexibility and elasticity, and their mechanical characteristics still do not meet clinical needs.

### Bone and cartilage tissue printing

Bone and cartilage regeneration is the most mature field utilizing printing technology because the composition of hard tissues is uncomplicated and is mainly composed of inorganic elements. A variety of biomaterials have been produced to construct bone and cartilage scaffolds by many manufacturing approaches, including gas foaming [84, 85], salt leaching [86, 87] and freeze drying [88, 89]. However, the structural and mechanical properties of artificial scaffolds can be more accurately controlled by 3D bioprinting than by other technologies.

A cement powder system was recently fabricated containing HA and TCP as the ideal composition for human bone replacement to repair large defects [90]. The dimensional accuracy of the bioprinted scaffolds was greater than 96.5 %. In Gao’s work [91], hMSCs and nanoparticles of bioactive glass (BG) and HA were co-printed to control the spatial placement of cells. hMSCs encapsulated in this compound exhibited high cell viability (86.62 ± 6.02 %) and compressive modulus (358.91 ± 48.05 kPa) after 21 days in culture. In Park et al. work [92], HA and Col-1 hydrogels printed as tissue-mimetic structures were investigated independently to elucidate their effects on the behavior of chondrocytes and osteoblasts. Chondrocytes on HA hydrogels and osteoblasts on Col-1 hydrogels maintained better proliferative capacity and cell function than chondrocytes on Col-1 hydrogels and osteoblasts on HA hydrogels.

Bioprinting has been a popular technology for the creation of cartilage tissue engineering scaffolds from a variety of materials, ranging from ceramics to nanomaterials. Markstedt et al. [77] developed a printable bioink using a combination of nanofibrillated cellulose and alginate with human chondrocytes as living soft tissue. The mixture exhibited excellent shear-thinning behavior and cell viability of 86 % after 7 days of 3D culture. The high plasticity of the ink allowed shapes resembling cartilage tissues, such as an ear and a meniscus, to be printed successfully.

Although printable scaffolds have been extensively applied in bone and cartilage tissue engineering, bioinks with suitable physical properties are still needed.

### Skin

Skin protects the body from attack by foreign substances and maintains the integrity of the body. Many chronic diseases and burn wounds cause irreversible damage to skin, and thus bioprinting technology is particularly critical in the preparation of skin substitutes for transplantation to repair damaged skin.

The LAB technique has been applied to the printing of bio-skin. Michael et al. [42] successfully created skin substitutes using LAB technology and transplanted them to skin wounds of nude mice. Eleven days later, the graft was able to adhere well to the tissue around the skin wound, and the cells in the graft were able to proliferate and differentiate. In 2014, Lee et al. used keratinocytes and fibroblasts as materials to bioprint skin tissue. The resulting skin tissue had representative morphology and biology. The skin bioprinted by 3D technology was able to maintain its shape and had high flexibility, reproducibility and culture throughput. In addition, cells that cause skin diseases can be added to the biomaterials, and skin tissue printed with pathogenic cells can be used to study the pathophysiology of skin diseases [93].

### Liver tissue printing

Compared to other organs, the liver has strong regeneration ability. Patients who require liver transplantation can receive lobes of liver from a healthy donor [94] or can also wait for their own liver tissue to regenerate. However, healthy donors are in short supply, and the regeneration period for self-liver tissue is long. Therefore, bioprinting of liver tissue by tissue engineering is particularly important.

Many researchers have studied liver bioprinting. Primary hepatocytes and stem cell-derived hepatocytes have been used as the bioink to bioprint liver tissue [95]. Although the cells lose a certain amount of activity and functionality in the printing process, liver tissue containing both cell types can be sustained for a period of time. In contrast to traditional printing technologies, 3D printing technology can provide the exact size and shape of the liver suitable for the needs of patients with liver resection [96]. Recently, a new technique has been used to maintain cell activity and functionality for longer times [97]. The technique utilizes bioprinting technology to form structures called “canaliculi” that are similar to the liver hepatic cord. The primary hepatocytes and the “canaliculus” structures are cultured together in the collagen matrix. Then, a biomimetic ECM system evaluates the activity and functionality of the primary hepatocytes on different ECM-based hydrogels [98]. After the activity and functionality of the primary hepatocytes have been confirmed, the cells can be maintained for 4 weeks. Further increases in cellular activity would facilitate expanded applications of bioprinted liver tissue. Chang et al. [99] also demonstrated that multilayered tissues can be used as in vitro 3D liver models. This multilayered tissue contained rat and human hepatocytes, and the multilayered cellular architecture could be used as a liver analog to help detect drug toxicities and for other medical and biological testing. Thus, 3D bioprinted livers can be used not only for liver resection in patients and other liver surgeries but also for simulated liver experiments in vitro.

The use of biological printing for the liver would have profound impact. Although some achievements have been made, hurdles must still be overcome, including cost and time, and the mechanical properties of printed liver tissue must be highly consistent with that of the native liver.

## Limitations of and future directions for bioprinting

Different types of 3D printing are utilized for applications that range from studies of cellular behavior to investigations of tissue pharmacodynamics or toxicological mechanisms. Although 3D printers have high precision and reproducibility, printing organs and functional tissues with entire structures still requires assembly layer-by-layer with “bio-glue.” The main technological barriers are suitable bioinks with good biocompatibility and mechanical strength that can be used to achieve biological function. Hydrogels and ceramics have been used for soft and hard tissue engineering, respectively [50, 55, 100]. Meanwhile, personalized 3D printing technology will lead to a series of regulatory hurdles referring to the specified printed product supervision. However, it is urgent for the management establishing and perfecting relevant laws and regulations to guarantee sustainable development of 3D printing technology. Studies in the near future will likely bring great progress in printing micro-organs, such as pancreas islet tissues that function in the absence of the complete pancreas structure, which will benefit hundreds of millions of diabetic patients around the globe. Chang [101] successfully fabricated micro-livers that were utilized for testing drug metabolism.

As printing technology develops, additional biomimetic, tissue engineered organs will be created. Decreases in reestablishment time and cost should also be addressed before bioprinting of organs can be applied in the clinic.

## Conclusions

Bioprinting technology has drawn more and more attention as a fabrication methodology for producing scaffolds, cells, tissues and organs. It has advantages in precise control, repeatability and individual design, yet many challenges remains for building complex tissues including multiple cell types in a spatial structure. More importantly, bioink materials development, resolution enhancement and vascularization are necessary to apply bioprinting technology clinically.

## Abbreviations

ECM: extracellular matrix
3D: three-dimensional
AM: additive manufacturing
CAD/CAM: computer-aided design/computer-aided manufacturing
CHO: Chinese hamster ovary
PAB: pressure-assisted bioprinting
MPSCs: multipotent stromal cells
LAB: laser-assisted bioprinting
SLS: selective laser sintering
PCL: polycaprolactone
HA: ydroxyapatite
TCP: tricalcium phosphate
CHAp/PLLA: hydroxyapatite/poly (l-lactic acid)
PVA: polyvinyl alcohol
STL: stereolithography
hMSCs: human mesenchymal stem cells
ESCs: embryonic stem cells
LIFT: laser-induced forward transfer
EGF: epidermal growth factor
FGF-2: fibroblast growth factor 2
FRESH: freeform reversible embedding of suspended hydrogels
